# Strengthened Default Mode Network Activation During Delay Discounting in Adolescents with Anorexia Nervosa After Partial Weight Restoration: A Longitudinal fMRI Study

**DOI:** 10.3390/jcm9040900

**Published:** 2020-03-25

**Authors:** Arne Doose, Joseph A. King, Fabio Bernardoni, Daniel Geisler, Inger Hellerhoff, Tomas Weinert, Veit Roessner, Michael N. Smolka, Stefan Ehrlich

**Affiliations:** 1 Division of Psychological and Social Medicine and Developmental Neuroscience, Faculty of Medicine, Dresden University of Technology, 01069 Dresden, Germany; 2 Translational Developmental Neuroscience Section, Eating Disorder Research and Treatment Center, Faculty of Medicine, Dresden University of Technology, 01069 Dresden, Germany; 3 Department of Psychiatry and Neuroimaging Center, Technische Universität Dresden, 01069 Dresden, Germany

**Keywords:** anorexia nervosa, eating disorders, delay discounting, intertemporal choice, reward processing, self-control, functional magnetic resonance imaging, longitudinal, functional connectivity, weight restoration treatment

## Abstract

The capacity of patients with anorexia nervosa (AN) to resist food-based rewards is often assumed to reflect excessive self-control. Previous cross-sectional functional magnetic resonance imaging (fMRI) studies utilizing the delay discounting (DD) paradigm, an index of impulsivity and self-control, suggested altered neural efficiency of decision-making in acutely underweight patients (acAN) and a relative normalization in long-term, weight-recovered individuals with a history of AN (recAN). The current longitudinal study tested for changes in functional magnetic resonance imaging (fMRI) activation during DD associated with intensive weight restoration treatment. A predominately adolescent cohort of 22 female acAN patients (mean age—15.5 years) performed an established DD paradigm during fMRI at the beginning of hospitalization and again after partial weight restoration (≥12% body mass index (BMI) increase). Analyses investigated longitudinal changes in both reward valuation and executive decision-making processes. Additional exploratory analyses included comparisons with data acquired in aged-matched healthy controls (HC) as well as probes of functional connectivity between empirically identified nodes of the “task-positive” frontoparietal control network (FPN) and “task-negative” default-mode network (DMN). While treatment was not associated with changes in behavioral DD parameters or activation, specific to reward processing, deactivation of the DMN during decision-making was significantly less pronounced following partial weight restoration. Strengthened DMN activation during DD might reflect a relative relaxation of cognitive overcontrol or improved self-referential, decision-making. Together, our findings present further evidence that aberrant decision-making in AN might be remediable by treatment and, therefore, might constitute an acute effect rather than a core trait variable of the disorder.

## 1. Introduction

Anorexia nervosa (AN) is a serious eating disorder characterized by extreme energy-intake restriction, significantly low body weight, intense fear of weight gain, and disturbances in body image [1]. The disorder typically develops in adolescence or young adulthood, affects women more often than men (8:1 ratio), frequently takes a chronic course, and has the highest mortality rate in psychiatry [2,3]. The unusual capacity of people with AN to resist hunger, postpone eating, and forgo food is often thought to be an expression of “too much” self-control [4,5]. Excessive self-control has long been recognized as an important clinical characteristic of AN [6], which together with altered reward sensitivity [7,8,9,10], might contribute to disorder maintenance and therapy resistance [11]. 

A growing number of studies have employed delay discounting (DD; or “intertemporal choice”) tasks in AN samples in an attempt to quantify reward-based decision-making in the disorder [12,13]. In a typical DD task, participants make a series of choices between an immediate monetary reward (smaller sooner, SS) and a larger one to be delivered after a certain delay (larger later, LL). DD tasks enable calculation of the individual discount rate, which specifies the degree to which a subjective reward value decreases as a function of the delay of delivery [14]. While steeper DD (i.e., a greater preference for immediate rewards) is generally taken to reflect more impulsive reward-related decision-making, shallower DD (i.e., greater propensity to delay gratification) is thought to indicate relatively self-controlled value-based choice [15]. 

Consistent with the notion of excessive self-control and an increased capacity to forgo immediate (food) rewards in the pursuit of long-term goals (thinness) in AN, several studies have reported shallower DD in acutely underweight patients (acAN), relative to healthy controls (HC) [16,17,18,19]. However, these observations were made in adults with a relatively long duration of illness and some studies have not detected group differences in discounting behavior [20,21], suggesting that a shallower DD might not reflect a defining trait variable of AN, no studies have found any altered DD in long-term weight-recovered AN (recAN) [20,22,23,24]. 

Functional magnetic resonance imaging (fMRI) studies indicate that two primary neurocognitive processes underlie DD: value-dependent processing and executive decision-making [25]. Value-dependent processing involves subjective reward valuation and activates brain regions including ventral striatum and ventromedial prefrontal cortex. Executive decision-making involves the actual choice process based on comparisons between alternative options, and is associated with activation of regions belonging to the frontoparietal control network (FPN), including lateral prefrontal cortex and posterior parietal cortex [26,27]. In AN samples, mixed findings from previous studies, including decreased reward-related activation in cingulostriatal circuitry in acAN [16] and increased activation in executive control regions in recAN [22], might be attributable to differences in study design, weight status, and analytic strategies [13]. Perhaps more important in relation to AN, which is often conceptualized as a disorder characterized by a fundamental deficit in self-regulation [28,29,30,31], intertemporal choices are inherently self-relevant [32] and DD is also commonly associated with activation changes in regions of the default mode network (DMN; including medial prefrontal cortex, posterior cingulate/precuneus, inferior parietal regions) [33] that are involved in self-reflection and prospection [34,35].

Disentangling state-related consequences of acute undernutrition (including drastic changes in the metabolome [36], the endocrine [37], cardiovascular systems [38], etc.) from trait factors is critical in AN research [4,39] and longitudinal observation can be particularly informative in this respect. To date, only two longitudinal studies of DD have been conducted in AN samples. In a behavioral pilot that we conducted neither altered DD in a predominately adolescent acAN sample nor any significant change in discount rates, following short-term weight restoration and cognitive-behavioral therapy [20] were observed. In contrast, Decker et al. [16] found normalization of a greater preference for delayed rewards (i.e., shallower DD), following a brief treatment, to be associated with abnormal fMRI activation in brain regions involved in both reward valuation and executive decision-making in adult patients. The current longitudinal study builds on our previous cross-sectional fMRI studies of DD which found both (1) faster and more consistent choice behavior and (2) decreased FPN activation, suggestive of altered neural efficiency of decision-making in acAN, relative to HC [40], and no evidence of either behavioral or neural alterations in recAN [23]. Although our previous behavioral study found no longitudinal changes in behavioral DD parameters [20], this does not preclude the possibility of meaningful changes in fMRI activation. Therefore, using the same experimental protocol, we tested here whether longitudinal changes in fMRI activation in predominately adolescent acAN, between study time point (TP1) at therapy admission and the second study time point after intensive treatment (TP2; minimum ≥ 12% body mass index (BMI) increase) would be more evident in brain regions involved in reward valuation (e.g., ventral striatum), executive decision-making (FPN), or self-referential processing (DMN).

## 2. Methods

### 2.1. Participants and Procedures

Twenty-six acutely underweight female patients diagnosed with AN according to the Diagnostic and Statistical Manual of Mental Disorders (DSM-IV) participated in the current longitudinal study. All participants were receiving treatment in a specialized eating disorder program. They were first assessed within 96 h, following treatment admission and beginning nutritional rehabilitation (TP1), and again after short-term weight restoration (TP2; ≥12% BMI increase). Although some patients reached a “normal” BMI at TP2, we considered all participants “partially weight-restored” given that it was unclear whether they would maintain this weight and the fact that the individual target weight which takes premorbid BMI into account is often above the epidemiological BMI cut-offs used in research. The ethics commission of the Technische Universitaet Dresden approved the study (EK 14012011, date: 06.02.2013) which was carried out in accordance with the Declaration of Helsinki. All participants (and their legal guardians) gave written informed consent. 

The diagnostic procedures, and the inclusion and exclusion criteria used for the current longitudinal sample were identical to those used for that in our previous behavioral pilot study [20] (see Supplementary Methods for detailed description). Pertinent information including potential confounding variables (e.g., medication, comorbidities) was obtained using the Structured Expert Interview for Anorexic and Bulimic Syndromes (SIAB-EX) [41], supplemented with our own semi-structured interview. Comorbid psychiatric diagnoses were confirmed by an experienced psychiatrist after consideration of the information collected from the clinical interviews, chart review, and the employed psychiatric screening instruments, which included the Eating Disorder Inventory–2 [42] and the Beck Depression Inventory–II [43]. Intelligence quotient (IQ) was estimated with short versions of the Wechsler Intelligence Scale for Children [44] or the Wechsler Adult Intelligence Scale [45]. BMI standard deviation scores (BMI–SDS) [46,47] were computed to provide an age-corrected index. Study data were managed using Research Electronic Data Capture [48].

The current longitudinal study was part of a greater research project on decision-making in AN. Data from participants at TP1 were published previously [40] (see Supplementary Methods). The data of 4 participants were excluded from further analysis, either due to abnormal choice behavior (choosing only larger later (LL) rewards or making >10% “illogical” choices (defined below): 3 datasets) or poor registration of the fMRI data (1 dataset); resulting in a final longitudinal sample of *n* = 22.

### 2.2. HC Participants

Supplementary exploratory comparisons were carried out with the data collected from randomly selected age-matched healthy controls (HCs) that participated in our previous cross-sectional studies ([20,40]; see Supplementary Materials). No longitudinal data were collected from HC.

### 2.3. Task

**Figure 1:**
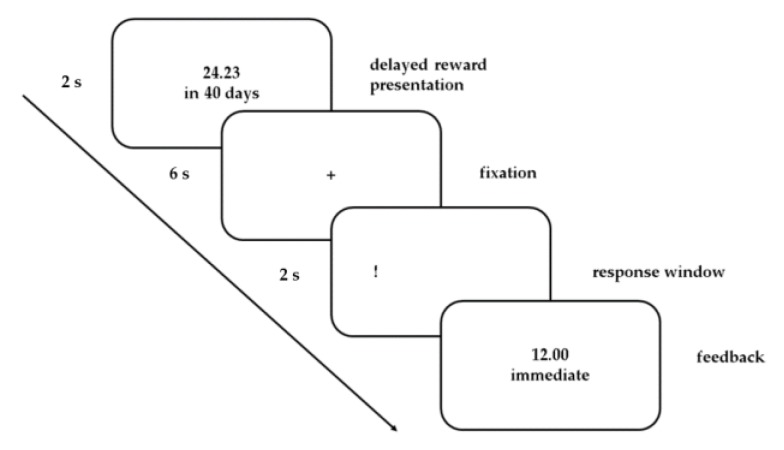
Main delay discounting functional magnetic resonance imaging (fMRI) task. Each of the 90 trials began with the presentation of the larger later (LL) reward (2 s), followed by a fixation cross (+; 6 s) and a response window (2 s). During the response phase, an exclamation mark (!) presented on the left or right side of the screen indicated whether the left or right button had to be pressed for the LL reward on that trial. Decisions for the LL reward were mapped to the right button in half of all trials and to the left button in the other half. Before the fMRI session began, participants were informed that they could choose the alternative smaller sooner (SS) reward (an individually determined fixed value between 5 € and 15 €, based on the individual discounting parameter k estimated in the pre-scan calibration session; see Supplementary Methods) by pressing the button on the opposite side. Feedback confirmed responses immediately. Between trials, a blank screen was presented for a jittered interval of seven seconds on average.

Participants performed the same two-part DD task as in our previous cross-sectional fMRI studies [23,40], at both time points. The task has been shown to demonstrate an ability to distinguish between age groups known to differ in self-control [49] and an overall good test-retest reliability, both on a behavioral and neural level in adolescents [50]. Participants first performed a pre-scan calibration session, including 50 choices between a fixed smaller sooner (SS) reward (20 €) or a LL one to be paid after a delay (10, 30, 60, 120, or 180 days). Based on these decisions, we calculated the individual discount parameter *k* as a metric of self-control and the amounts and delays of rewards in the main fMRI task were adapted accordingly (For further details regarding the calibration task, see Supplementary Methods). Immediately following the calibration session, participants performed the main task (Figure 1) during fMRI. Before the fMRI task began, participants were informed about the SS reward (fixed value between 5 € and 15 €; mean = 9.4, standard deviation (SD) = 2.8), which was determined based on the individual discounting parameter *k*. The delay times were the same as in the calibration session. Pairs of reward amounts and delays were calculated in advance and presented randomly across the task (sample pairs are presented in Supplementary Table S1). To encourage realistic decisions, participants were informed that one of their choices would be selected randomly and the reward would be paid either immediately after the experiment (for a SS choice) or following the chosen delay by bank transfer (for a LL choice). The task was programmed using the Presentation^®^ software (version 16.1, Neurobehavioral Systems, Inc., Berkeley, CA, USA).

### 2.4. Behavioral Data Analysis

Behavioral data were analyzed as in our previous cross-sectional fMRI studies in AN [23,40] and originally described in Ripke et al. [49]. For comparison of the discount parameter *k* estimated at TP1 and TP2 (paired-samples *t*-test), we used log-transformed values because they better fit a normal distribution. We gauged the quality of decision-making during the main fMRI experiment by calculating area under the curve (AUC) as a consistency metric. AUC reflects the degree at which participants opted for the subjectively more valuable reward option and is higher for more consistent decision-making (i.e., always choosing the reward with the higher subjective value results in an AUC of 1; complete randomness of choices would yield an AUC of 0.5). A difference in AUC between TP1 and TP2 was tested (paired-samples *t*-test) using rank-transformed values that better fit normal distributions. Trials with illogical choices (defined as decisions for a reward whose subjective value was lower than half of the alternative option [49]) and those without a response were regarded as invalid and excluded from all analyses. As in our previous fMRI studies [23,40], an additional behavioral measure was mean reaction time (RT) on the valid trials of the main fMRI session. RT data were analyzed with a 2 (chosen reward: SS vs. LL) × 2 (time point: TP1 vs. TP2) repeated-measures ANOVA.

### 2.5. FMRI Acquisition, Processing, and Analysis

Image acquisition (Siemens (Erlangen, Germany) 3T MRI Scanner; 32-channel head coil) was analogous to our previous fMRI studies [23,40]. All scanning was conducted between 8 and 9 AM following an overnight fast, to control for metabolic state and circadian processes. 

First, a structural T1-weighted magnetization-prepared rapid acquisition with gradient echo (MPRAGE) sequence (TR = 1900 ms, TE = 2.26 ms, FOV = 256 × 256 mm, 176 slices, 1 × 1 × 1 mm^3^ voxel size, flip angle = 9°) was run. For functional imaging, a gradient-echo T2*-weighted EPI sequence (TR = 2410 ms, TE = 25 ms, flip angle = 80°) was used. A total of 636 volumes (42 transversal slices orientated 17° clockwise to the anterior commissure *-* posterior commissure (AC-PC) line, 2 mm slice thickness, 1 mm gap, OV = 192 × 192 mm, in-plane resolution of 64 × 64 pixels = voxel size of 3 × 3 × 2 mm^3^) were acquired. Behavioral data were recorded with NordicNeuroLab response grips (NordicNeuroLab, Bergen, Norway).

Imaging data were processed and analysed using SPM8 ((Wellcome Department of Cognitive Neurology, Institute of Neurology, Queen Square, London)) within the Nipype framework [51], as in our previous studies [23,40]. Functional images were first slice-time corrected, realigned, and registered to their mean. The six realignment parameters were later used as covariates in the first level analysis to correct for rigid body movement. The realigned images were coregistered to the participant’s structural image. Images were manually quality controlled and volumes with signal intensity outliers (>3 SD from the time-series mean) or excessive movement (>2 mm in each direction), as determined by artefact detection tools (ART) were discarded (mean 14 volumes per participant). All participants’ structural images were used to generate a sample-specific DARTEL template [52]. This template was used to normalize the functional volumes to the Montreal Neurological Institute (MNI) standard space. Finally, images were smoothed with an isotropic Gaussian kernel (8 mm FWHM). 

First-level statistical analysis was based on least-squares estimation using a GLM, as in previous studies that employed the task [23,40,49,50,53]. The main model consisted of the two regressors of primary interest—(1) onsets of delayed rewards, (2) subjective value of the delayed reward as a parametric modulator of regressor 1. The model additionally included the onsets of the response–feedback phase, invalid trials, and 6 realignment parameters, and outlier volumes as nuisance regressors. All events were modeled as stick functions (zero duration), convolved with the canonical synthetic HRF. In this model, regressor 1 was assumed to gauge activity generally associated with executive decision-making during DD, while the parametric regressor 2 represented the correlation of the decision-making activity with its subjective value. To confirm the expected patterns of increased activation in the regions associated with executive decision-making (e.g., lateral prefrontal cortex and posterior parietal cortex) and reward valuation (e.g., ventral striatum and ventromedial prefrontal cortex), based on previous studies with our task, we first ran two separate exploratory one-sample *t*-tests, for regressors 1 and 2, respectively. Our main whole-brain analyses then tested for longitudinal changes (TP1 vs. TP2) in each of these processes with second-level paired *t*-tests. As in our previous studies [23,40], we also tested for potential differences in value-dependent processing between time-points by estimating an additional GLM with separate regressors for SS and LL decisions, followed by a paired *t*-test of the SS vs. LL contrast. We controlled for false-positive results in whole-brain analyses with 3DClustSim (), as in our previous studies [23,40].

An exploratory repetition of the acAN-TP1 vs. HC comparison (as in our previous cross-sectional study [40]) based on 22 age-matched participants included in the current study is described in the Supplementary Table S2. Based on our previous findings that were suggestive of an altered activation of the dorsal anterior cingulate cortex (dACC) as a function of decision difficulty in acAN-TP1 [40] and a relative normalization thereof, after long-term weight restoration [23], we also carried out a region of interest (ROI) analysis of longitudinal changes in dACC activity (extracted with MarsBaR toolbox [54]) associated with easy and hard decisions, using the same methods employed in the previous studies (Supplementary Methods). Additional exploratory analyses tested for correlations between activation in empirically-defined ROIs and changes in relevant clinical variables (scores of questionnaires listed in Table 1) and DD parameters (*k* and AUC) between time-points. Finally, in an attempt to gain further insight into the underlying nature of alterations in the neural correlates of decision-making during DD in AN, we conducted a series of functional connectivity analyses using an ROI-to-ROI approach implemented in the CONN toolbox [55] between regions in which we previously observed group differences, relative to HC [40] and those identified in the current longitudinal study (see Supplementary Methods).

## 3. Results

### 3.1. Behavioral Results

Demographic and clinical characteristics are summarized in Table 1. Although the values do not show complete symptom normalization, increased BMI after weight restoration therapy at TP2 and a substantial clinical improvement were evident in significantly decreased eating disorder and general psychiatric symptoms.

**Figure 2:**
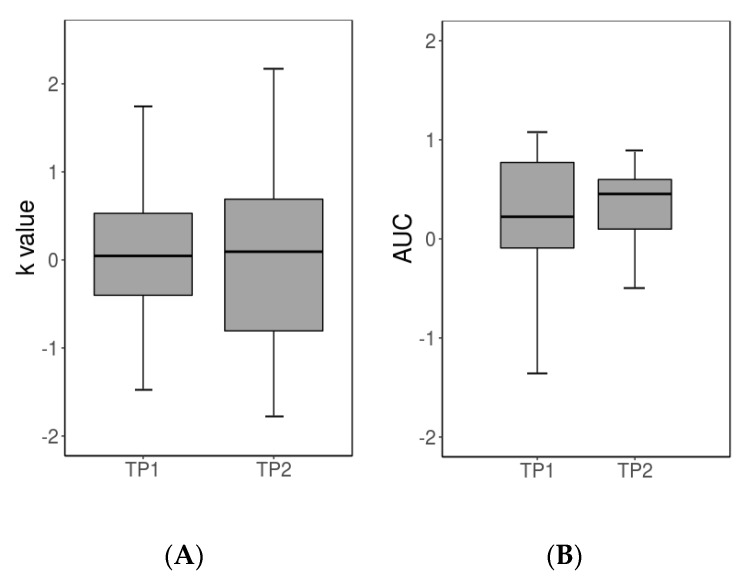
Behavioral results from the delay discounting paradigm. (**A**) Boxplot of logarithmic and z-standardized k values estimated from the choices of the pre-scan calibration session at TP1 (mean raw value = 0.009; SD = 0.006) and TP2 (mean raw value = 0.008; SD = 0.007). (**B**) Boxplot of z-standardized AUC values estimated from the choice behavior, during the main task at TP1 (mean raw value = 0.775; SD = 0.065) and TP2 (mean raw value = 0.796; SD = 0.054). In both panels, the thick black line indicates the median. The lower/upper ends of the vertical line indicate the minimum/maximum values, while the box represents the first to third quartiles.

In line with the longitudinal observations of our behavioral pilot study [20], no significant difference between mean individual discounting rates estimated at TP1 and TP2 was detected (T_21_ = 0.00; n.s.; Figure 2(A)). Similarly, regarding the consistency at which participants chose rewards with a higher subjective value, AUC values estimated at TP1 (0.91) did not clearly differ from those at TP2 (0.95; Wilcoxon rank-sum test; *p* = 0.057; Figure 2(B)). As in our previous cross-sectional fMRI studies [23,40], mean reaction times were generally faster for the LL decisions than the SS decisions (622 ms vs. 670 ms; F_1,43_ = 51.2; *p* < 0.05). However, they did not generally differ between time points (TP1 641 ms vs. TP2 651 ms; F_1,43_ = 2.1; n.s.) and no interaction between choice and time-point was evident (F_1,43_ = 0.02; n.s.).

### 3.2. Imaging Results

Exploratory whole-brain analyses inspecting the main effects of (1) executive decision-making (regressor 1) and (2) value-dependent processing (regressor 2), confirmed the expected activation patterns elicited by the employed task (see Supplementary Figure S1).

**Figure 3:**
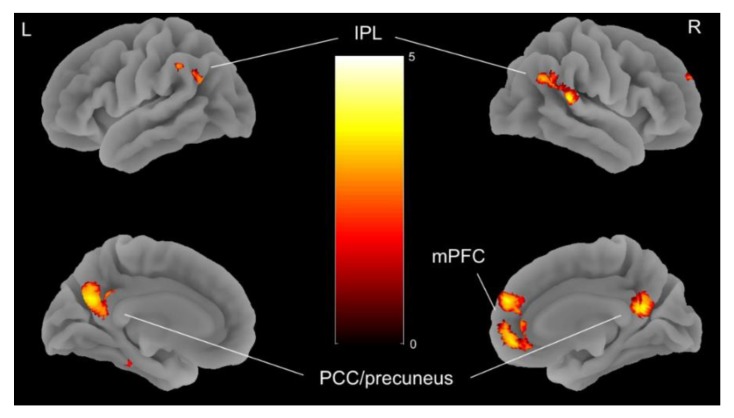
Family-wise error (FWE) rate corrected (*p* < 0.05) brain map showing regions in which blood-oxygen-level-dependent (BOLD) signal deactivation associated with executive decision-making during delay discounting (regressor 1) was less pronounced in AN, following partial weight restoration (i.e., time point 2 (TP2) > time point 1 (TP1)), indicating a relative activation increase. *T*-map values are plotted on a 3 dimensional (3D) surface rendering of an individual brain normalized to the Montreal Neurological Institute template. AN = anorexia nervosa; mPFC = medial prefrontal cortex; IPL = inferior parietal lobe; PCC = posterior cingulate cortex. L = left; R = right.

More importantly, no activation differences between TP1 and TP2 in the parametric analysis targeting value-dependent processing (i.e., regressor 2; or in an exploratory analysis of the basic SS vs. LL contrast) survived the correction for multiple comparisons. However, activation associated with executive decision-making (regressor 1) both decreased and increased in several regions. Specifically, activation decreased following partial weight restoration in a region of right middle occipital cortex and increased in bilateral DMN regions, including the medial prefrontal cortex, posterior cingulate/precuneus, and inferior parietal lobule (Table 2; Figure 3). These so-called “task-negative” DMN regions in which activation increased from TP1 to TP2 (i.e., showed less deactivation) are typically deactivated as a function of cognitive demand [56], such as during DD [25], and anticorrelated with activation in so-called “task-positive” regions, including the FPN [57]. Indeed, exploratory functional connectivity analyses confirmed anticorrelated activity between the identified DMN regions and FPN regions that were generally recruited during DD (regressor 1; Supplementary Figure S2), but no longitudinal effects were observed. Additional supplementary analysis suggested that the relative attenuation of DMN deactivation at TP2 could be partially, albeit minimally, attributed to interindividual differences in weight gain and time between TP1 and TP2.

**Figure 4:**
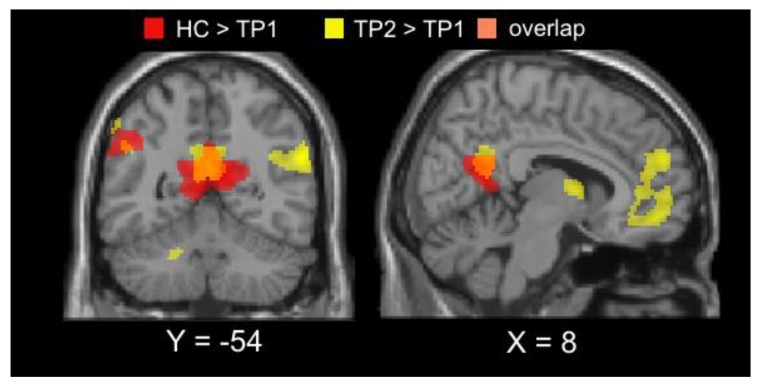
Qualitative comparison between (1) longitudinal changes in default mode network (DMN) deactivation associated with executive decision-making during delay discounting (regressor 1), following partial weight restoration (yellow regions) and (2) cross-sectional group differences between acAN-TP1 and HC (red regions) to illustrate their overlap (orange regions). The respective t-maps were thresholded at *p* < 0.005 (uncorrected) for illustrative purposes. Note that while the cross-sectional group differences depicted in the figure were based on data limited to the acAN-TP1 participants in the current longitudinal study, they largely mirrored the results of the King et al. [40] in a larger sample. acAN-TP1 = acutely underweight anorexia nervosa-time point 1; HC = healthy control.

Additional supplementary comparisons between the acAN-TP1 sample included in the current longitudinal analyses and HC, confirmed stronger DMN deactivation in patients during executive decision-making (regressor 1), as observed in our previous cross-sectional study [37] (Figure 4; Supplementary Figure S3). Exploratory comparison of acAN-TP2 and HC did not reveal any significant differences between groups, similar to the results presented by King et al. [23] in long-term, weight-recovered AN and HC.

**Figure 5:**
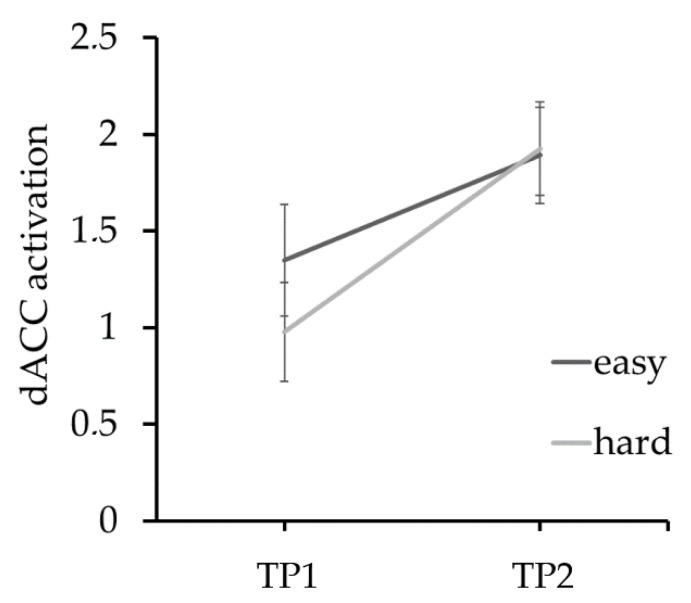
Mean activation (β estimates ± standard error of the mean) in the dACC region of interest identified in King et al., [40] on trials with easy and hard decisions in AN patients at TP1 and TP2. A time point × decision difficulty (hard/easy) repeated-measures ANOVA of the β estimates indicated that while activation was generally increased in this region at TP2 (main effect of time point; F_1,43_ = 8.2; *p* < 0.05), the typical increase of activation in hard vs. easy trials observed in both HC and long-term weight recovered AN patients [23,40] and several other studies (for review, see Koffarnus et al. [59]) was absent (main effect of decision difficulty; F_1,43_ = 0.4; n.s.). Further, no time point × decision difficulty interaction was evident (F_1,43_ = 0.6; n.s.). AN = anorexia nervosa; dACC = dorsal anterior cingulate cortex; HC = healthy control; TP1 = time point 1; TP2 = time point 2.

In our previous study of acAN-TP1, we interpreted reduced FPN activation during DD as a potential neural maker of altered efficiency of decision-making in the disorder [40]. This hypothesis was supported by the consistent faster choice-behavior of patients and decreased activation for hard vs. easy decisions in the dorsal anterior cingulate cortex (dACC); a region involved in resolving conflict between competing decisions [58] and commonly activated as a function of decision difficulty (hard > easy) in DD tasks [59]. Longitudinal analysis of activation in the dACC ROI identified by King et al. [40] revealed a general increase at TP2 (Figure 5). However, in contrast to dACC response to decision difficulty in both long-term, weight-recovered AN [23] and HC, activation in this region was still undifferentiated between hard vs. easy decisions at TP2 (Supplementary Figure S4).

Exploratory analysis of potential correlations between activation in regions in which significant changes in activation between TP1 and TP2 were observed (Figure 3, Table 2) and changes in relevant clinical characteristics and DD parameters, did not reveal any statistically significant relationships (all *p*_FWE_ > 0.4).

## 4. Discussion

This longitudinal study investigated changes in the neural substrate underlying DD in a sample of adolescent acAN patients undergoing intensive weight restoration treatment (≥12% BMI increase). In line with our previous behavioral pilot study [20], we found no longitudinal changes either in the DD rate or the consistency or in the speed of decision-making, after partial weight restoration. We also found no longitudinal changes in fMRI activation associated with subjective reward valuation (regressor 2). When considered in conjunction with our previous cross-sectional findings that were suggestive of relatively intact value-dependent processing in both acAN and recAN as compared to HC [23,40], this finding delivered further evidence that monetary DD tasks such as ours might not be well-suited to capture clinically-relevant alterations, specific to reward sensitivity in AN. Importantly, however, the fMRI analysis targeting brain regions involved in executive decision-making (regessor 1) revealed less deactivation in the core nodes of the “task-negative” DMN at follow-up relative to baseline (TP2 > TP1). This finding is relevant not only to the literature on reward-based decision-making (including DD) in AN [7,60,61], but also to the greater functional neuroimaging literature in the disorder [10,62,63,64], for at least three reasons. First, the “task-negative” DMN regions in which longitudinal changes were observed (medial prefrontal cortex, posterior cingulate/precuneus, inferior parietal lobule) highly overlapped those in which we previously found abnormally decreased activation (i.e., greater deactivation) in acAN-T1, in comparison to HC, using the same analysis [40]. Thus, given the known anticorrelation between activation in the DMN and that in “task-positive” regions including the FPN [57,65] (also demonstrated here in the current data; see Supplementary Figure S2), increased DMN activation (i.e., less deactivation) after partial weight restoration is suggestive of a relative normalization of neural activity associated with the actual decision-making process during DD. The likelihood of this normalization was supported by the absence of significant differences between acAN–TP2 with a matched HC sample in an exploratory analysis (regressor 1). However, suggesting that normalization was only partial and regionally specific, dACC activation generally increased in acAN–TP2, but still did not exhibit a normal response on difficult vs. easy trials [59], as observed previously in both HC and recAN [20,40]. Second, while aberrant DMN function has been repeatedly described in AN, both at rest [66,67] and during disorder-relevant stimulation [68,69,70,71,72], the apparent longitudinal improvement observed here in a task that recruited the FPN, suggests that the abnormalities might be primarily associated with the underweight state and might not constitute a trait variable of the disorder. Based on our previous findings of consistently faster decision-making and altered neural efficiency in the FPN during DD in acAN compared to HC [40], we speculate that the relative attenuation of DMN deactivation in acAN-T2 might reflect a relaxation of excessive self-control over the course of treatment or a shift from more habitual to intentional execution of self-control. However, the longitudinal activation increase in visual (middle occipital) cortex suggestive of greater task focus at follow-up, might speak against this interpretation and alternative hypotheses should also be considered. Given the self-relevant nature of intertemporal choices [32,73], the involvement of DMN in self-referential processing [74,75] and the notion that AN is characterized by fundamental disturbances in self-identity [28,29,30], another interpretation of increased DMN activation after weight restoration is that it might reflect improved self-regulation and the ability to integrate self-relevant signals in decision-making. The likelihood of this alternative account is supported by findings of differences in DMN activation in AN samples in various tasks involving self-perception and evaluation [69,70,71,72]. Last but not the least, regardless of which interpretation best fits the data, the observed longitudinal activation changes in a task with good group-level test-retest reliability [50] underlines the notion that DD might not be a static trait, as is often thought [76,77], but is rather a highly malleable and task-dependent process [78].

Comparing the current findings in a predominantly adolescent sample with those of the only other known investigation of longitudinal neural changes in DD in adults [16] is difficult not only because of potential (neuro-)developmental differences [49,79], but also the task-dependent nature of both the behavioral and neural correlates of DD [25,35,78]. It is nonetheless noteworthy that Decker et al. [16] found, similar to the current study, normal discounting behavior post-treatment and a generally increased activation in all regions in which group differences relative to HC were evident. However, while the longitudinal activation increases observed by Decker et al. [16] were located in circuitry implicated in reward processing and executive decision-making, those that reached significance in the current study belonged primarily to the DMN. More research is needed to clarify these partially discrepant results. To disambiguate between the alternative interpretations offered above, future studies might employ DD paradigms in which intertemporal choices are made both for the self and for others [80].

When considering the findings discussed above, some important study limitations need to be taken into account. First and foremost, we cannot definitively exclude the possibility that the changes observed at acAN-T2 might rather reflect retest effects given the lack of a longitudinal HC group. However, given the nature of the task and the fact that the time between the two assessments was on average > 3 months, potentially confounding “training” effects should be small. Another shortcoming might be a lack of statistical power due to the relatively few number of participants. Nonetheless, it is also important to note that our longitudinal sample is the largest in this particular field of research to date. Further, it remains unclear how treatment-related changes in DMN activation during DD might be related to the observed clinical improvements as no correlations were found. As previously implied by Decker et al. [16], however, the lack of correlation between the measures of DD and the clinical variables might be partially attributable to the monetary rewards used in our paradigm and future research, including disorder-relevant stimuli [64], which might be more informative in this respect [12]. Last but not the least, differentiating between state and trait effects is one of the challenges in AN research. Although studying patients after both short- and long-term recovery can help to address this question, it is difficult to exclude persisting secondary effects from long periods of undernutrition—so called “scars”. However, studying adolescents (with a short duration of illness) might help to minimize such bias.

A better understanding of the neural mechanisms underlying maladaptive choice behavior in AN is important to the development of effective treatments [62,81,82]. The current findings make at least two contributions in this effort. First, they deliver evidence suggestive of a partial normalization of neural processes subserving DD, after short-term weight restoration. The longitudinal changes in DMN activation observed here during performance of a DD task dovetail with previous findings of alterations in these regions in individuals with AN, both at rest [66,67] and during other self-relevant decision-making tasks [69,70,71,72]. Although supplementary analyses of functional connectivity between the identified DMN regions and task-positive FPN regions did not detect significant longitudinal differences, future-focused analysis of antagonism between task-positive and task-negative networks [83] might provide a unique window on brain function and dysfunction in AN [84,85,86]. Second, our findings also provide further evidence underlining the volatility of DD [78] and suggest that alterations in AN likely do not constitute a trait of the disorder, as might be inferred from recent meta-analytic data [19,24].

## Figures and Tables

**Table 1:** Demographics and clinical characteristics of the longitudinal anorexia nervosa (AN) sample (N = 22).

	TP1	TP2	t	*p*
Age (years)	15.5 ± 2.2	15.8 ± 2.2	−11.7	<0.001
IQ	114 ± 11.1	-	-	-
BMI-SDS	−2.9 ± 1.0	−0.7 ± 0.6	−13.7	<0.001
BMI (kg /m²)	14.9 ± 1.2	18.8 ± 1.2	−15.3	<0.001
Duration of current episode (months)	11.6 ± 16.8	-	-	-
BDI-II	20.0 ± 9.5	12.1 ± 6.4	4.4	<0.001
SCL-90-R (Global Severity Index)	0.9 ± 0.5	0.6 ± 0.4	4.3	<0.001
EDI-2 total	203.6 ± 38.6	188.3 ± 41.8	2.4	0.03
EDI-2 Drive for thinness	30.1 ± 8.5	25.5 ± 10.5	2.2	0.04
EDI-2 Body dissatisfaction	36.5 ± 10.3	37.5 ± 13.3	−0.3	0.75
EDI-2 Bulimia	10.1 ± 2.9	8.5 ± 1.9	3.01	0.01

Time between TP1 and TP2 (≥12% BMI increase); mean = 101 days and SD = 41 days. Data are presented as mean ± standard deviation. All patients were of the restrictive AN subtype, as ascertained with the SIAB-EX interview. Two patients were formally diagnosed with comorbid psychiatric disorders (1 major depression, 1 illness anxiety/somatic symptom disorder). BDI-II = Beck Depression Inventory–II; BMI = body mass index; BMI-SDS = body mass index standard deviation scores; EDI-2 = Eating Disorder Inventory–2; IQ = Intelligence quotient and SCL-90-R = Symptom Checklist-90–Revised.

**Table 2:** Activation differences in executive decision-making (regressor 1) between TP1 and TP2.

	Hemisphere	Voxels	x	y	z	t_max_
TP1<TP2						
Middle occipital gyrus	L/R	495	22	−98	14	6.05
TP1>TP2						
Angular gyrus	L	368	−54	−68	32	−4.35
Angular gyrus	R	582	56	−56	25	−5.60
Posterior cingulate/precuneus	L/R	754	6	−54	20	−4.67
Medial Frontal Gyrus	L/R	600	8	54	26	−4.60

*p*_FWE_ <0.05. AN = anorexia nervosa; FWE = familywise error; HC = healthy control; L = left; R = right.

## Supplementary Materials

The following are available online at https://www.mdpi.com/2077-0383/9/4/900/s1, Table S1: Exemplary sample of absolute amounts offered during the experiment and respective subjective values. Table S2: Demographic variables and clinical characteristics of AN at TP1 and age-matched HC included in exploratory cross-sectional analyses (complete sample, N = 22 in each group). Figure S1: BOLD activation patterns for “value-dependent” (regressor 2) and “value-independent” (regressor 1) contrasts. Figure S2: Exemplary 3D plot of the FDR corrected ROI-to-ROI analysis. Figure S3: Results of exploratory group comparisons between the acAN-TP1 included in the current longitudinal study (*n* = 22) and age-matched HC (*n* = 2 2). Figure S4: Longitudinal analysis of activation in the dACC ROI identified by King et al. [40].
