# Supplementary material for: Strengthened Default Mode Network Activation During Delay Discounting in Adolescents with Anorexia Nervosa After Partial Weight Restoration: A Longitudinal fMRI Study
Source: J Clin Med. 2020 Mar 25;9(4):900. doi: 10.3390/jcm9040900 (PMC7230250; doi:10.3390/jcm9040900)
Supplement: Supplementary file 1 [file jcm-09-00900-s001.pdf]

Supplementary Materials

Supplementary Methods

Participants

Diagnosis of AN participants was established as described previously [20,23,40] using a semistructured research interview that requires a body mass index (BMI) lower than the 10th age percentile (if age<15.4 year) or a BMI lower than 17.5 (if age > 15.5) as well as no psychotropic medication (within 6 weeks prior to the study).

For the follow-up analysis of HC versus acAN-TP1/TP2 an automated search algorithm for optimal pairs [84] was used for age-matching. The inclusion criteria for HC participants correspond to King et al. [40] and Ritschel et al. [20]. HC participants were excluded, if they had a lifetime body mass index (BMI) lower than the 10th percentile (for participants under the age of 18 years) or below 18.5 kg/m² (age > 18 years) or were obese. Demographic characteristics of the HC group are summarized in Table S2. Further, any lifetime clinical diagnosis including organic brain syndrome, schizophrenia, substance dependence, psychosis not otherwise specified (NOS), bipolar disorder, bulimia nervosa or binge-eating disorder (or 'regular' binge eating, defined as bingeing at least once weekly for ≥3 consecutive months) was an exclusion criteria for all participants. All participants had no current inflammatory, neurological or metabolic illness, and had no chronical medical, neurological or metabolic illness with known effects on body weight or eating behavior, clinically relevant anemia, pregnancy or were breastfeeding. All participants had an intelligence quotient higher than 85 (IQ>85).

Task

For the calibration phase 5 blocks of 10 trials with varying delay times were shown. Each delay time was presented in blocks of 10 trials, after which the delay time changed, e.g. 10 trials with a delay of 10 days and varying monetary offers.

Calculation of delay discounting parameter k

Calculation of the individual delay discounting parameter k was identical to that in our previous studies [23,40] and originally described by Ripke et al., [52]. Based on the choices in the calibration phase, the indifference amount was calculated for each delayed option. The indifference amount is the mean of the maximum rejected delayed amount and the selected minimum delayed amount. This results in a point described by value and delay. The combination of these points for each delay time can be described using a hyperbolic function.

Equation (1)

$$V_d = \frac{A}{1 + k \times D}$$

The function describes how the subjective value V_d of the indifference amount A is reduced by the delay duration in relation to the individual discounting parameter k. The discounting parameter k was estimated to best fit the hyperbolic function described by the six points consisting of the 20 Euros for immediate reward (delay=0 days) and five indifference amounts for each delay (10, 30, 60, 120,180).

Based on the decisions in the calibration phase, the individual discount parameter k was calculated which was the primary behavioural variable of interest. A higher individual k value describes the participants' preference for an immediate reward over a future reward even though the future reward has a higher relative value. The amounts and delays of options of the main fMRI task were calibrated

to the individual discount parameters. The goal was to present offers so that the participants choose the immediate reward/delayed reward each in 50% of the cases. Second, the mean value of all rewards chosen should be roughly the same (30 Euro) for each of the participants. Third, the maximal reward (*V*) should be twentyfold the minimal reward offered. All offers for each subject were calculated individually in advance. For each delay time nine rewards higher and nine lower than the individual immediate reward were calculated using the following Equation (2):

Equation (2)

$$V_d = V_0 \frac{1 + k \times D \times c}{1 + k \times D}$$

V_d represents the subjective values of the delayed options (LL) while V_0 represents the subjective value of the immediate option (SS). The parameter *c* ensures the SS option to be always lower than the LL option and was set to 0.1, 0.15, 0.20, 0.25, 0.30, 0.35, 0.40, 0.45, and 0.50. To guarantee the range of possible choices was the same for every subject, the maximum reward *V* for each delay was twentyfold the smallest reward for 180 days delay (*D*). The distance between maximum reward and immediate reward was divided into nine equidistant miniblocks each consisting of 10 trials with a value above the individual immediate reward.

Decision difficulty ROI analysis

As in our previous studies [23,40], we estimated an additional GLM to investigate activation associated with decision difficulty. Decisions were categorized as "hard" if the ratio between the value of the immediate reward and the subjective value of the delayed reward was proximate to the individual indifference point and "easy" if they were distant. Specifically, we sorted trials according to this ratio so that choices that fell between the 25th and the 75th percentile were classified as hard, while the rest were classified as easy. To test for potential longitudinal changes activation associated with decision difficulty, we submitted beta estimates extracted for easy and hard trials from the dACC volume identified by King et al. [40] (x −10, y −30, z 28; 580 voxels) using MarsBaR toolbox (http://marsbar.sourceforge.net/) to 2 (time point) × (difficulty) repeated measures ANOVAS.

Connectivity analyses

ROI-to-ROI analyses were implemented using the CONN toolbox within SPM 12. Preprocessed data were band-pass filtered and white matter and cerebrospinal fluid were used as confounding variables following the implemented CompCor [85] correction feature. Bi-variate Fisher-transformed correlations of ROIs (extracted with MarsBaR toolbox (http://marsbar.sourceforge.net/)) identified in the main experiment (see Table 2 in the main article) were calculated on an individual level for all subjects, corrected for multiple comparison and used for a second level paired-sample t-test between time points.

Supplementary Results

Exploratory one-sample t-tests of the fMRI data confirmed expected patterns of hemodynamic activity associated with value-dependent processing (regressor 2) and executive decision making (regressor 1) as in our previous study [40] and the original studies using the employed task [49,52]. Specifically, correlation between the subjective value of chosen rewards and BOLD activity was clearly evident in regions thought to be involved in processing appetitive stimuli including e.g. the ventral striatum/midbrain and the rostral anterior cingulate cortex/ventromedial prefrontal cortex and elevated activation was present in executive control regions commonly recruited during deliberation between alternative options including lateral prefrontal cortex, posterior medial frontal cortex (including the dorsal anterior cingulate cortex and pre-supplementary motor area) and posterior parietal cortex during intertemporal choice (Figure S1).

Additional exploratory cross-sectional comparisons between AN and age-matched HC (two-sample t-tests) returned significant results in the executive decision-making contrast largely mirroring those previously found in the greater acAN-TP1 vs HC sample of King et al. [40] (Figure S3), but no group differences reached significance in any acAN-TP2 vs HC comparisons.

Additional supplementary analysis sought to verify the potential influence of the degree of BMI change and the amount of time between TP1 and TP2 on our main findings of a longitudinal attenuation of DMN deactivation. To this end, we submitted activation parameter estimates (betas averaged across all significant voxels) extracted from both the whole observed DMN network (i.e. all regions shown in Figure 3 in the main article) and each of the identified regions individually (mPFC, precuneus/PCC, bilateral IPL) to two separate repeated-measures ANCOVAs with 1. BMI-SDS change and 2. days between scans as covariates. The main effect of timepoint remained significant both in the whole DMN network and in each of the individual nodes (all $F > 4.4$; all $p < .05$) except for the precuneus/PCC where the difference in activation between timepoints was nudged to trend level after inclusion of BMI-SDS change ($F = 3.8$; $p = .057$) and days between scans ($F = 3.7$; $p = .059$).

Table S1: Exemplary sample of absolute amounts offered during the experiment and respective subjective values. Data for this hypothetical participant was generated with a hypothetical discounting value of k= 0.0084. SS reward was set to 10 Euro.

10 days		30 days		60 days		120 days		180 days	
amount offered	subjective value	amount offered	subjective value	amount offered	subjective value	amount offered	subjective value	amount offered	subjective value
60.13	53.70	46.70	34.35	42.42	24.67	17.18	7.04	19.77	6.26
12.36	11.04	99.32	73.05	142.23	82.72	154.55	63.37	169.58	53.70
12.72	11.36	13.94	10.25	15.88	9.24	201.74	82.72	14.59	4.62
38.47	34.35	13.29	9.78	92.33	53.70	18.04	7.40	108.48	34.35
92.64	82.72	12.86	9.46	59.06	34.35	14.59	5.98	291.80	92.39
70.97	63.37	14.16	10.41	75.69	44.02	107.36	44.02	21.06	6.67
27.63	24.67	12.43	9.14	108.96	63.37	130.96	53.70	47.37	15.00
49.30	44.02	33.55	24.67	15.02	8.74	178.14	73.05	200.14	63.37
12.43	11.10	125.63	92.39	12.86	7.48	19.77	8.11	18.47	5.85
103.47	92.39	12.65	9.30	15.45	8.99	15.45	6.34	139.03	44.02
81.80	73.05	13.51	9.94	158.86	92.39	36.58	15.00	17.18	5.44
12.50	11.16	86.17	63.37	16.32	9.49	83.77	34.35	24.95	7.90
12.29	10.97	73.01	53.70	14.16	8.23	13.73	5.63	23.65	7.49
12.22	10.91	20.40	15.00	25.79	15.00	16.32	6.69	77.93	24.67
12.65	11.29	59.86	44.02	14.59	8.49	20.63	8.46	230.69	73.05
16.80	15.00	13.73	10.10	13.29	7.73	18.91	7.75	261.24	82.72
12.58	11.23	13.08	9.62	13.73	7.98	60.18	24.67	22.36	7.08
12.14	10.84	112.47	82.72	125.59	73.05	225.33	92.39	15.88	5.03

Table S2: Demographic variables and clinical characteristics of AN at TP1 and age-matched HC included in exploratory cross-sectional analyses (complete sample, N=22 in each group).

	**TP1**	**TP2**	**t**	***p***
Age (y)	15.5 ± 2.2	15.5 ± 2.3	0.05	0.96
IQ	114 ± 11.1	113 ± 6.5	0.48	0.64
BMI (kg /m^2)	14.9 ± 1.2	20.3 ± 2.1	-10.60	< .001
BMI-SDS	-2.9 ± 1.0	-0.0 ± 0.7	-11.07	< .001
Minimum lifetime BMI	14.3 ± 1.38	19.2 ± 1.4	-12.10	< .001
BDI-II	20.0 ± 9.5	7.2 ± 5.9	5.36	< .001
SCL-90-R (Global Severity Index)	0.9 ± 0.5	0.4 ± 0.4	3.42	< .001
EDI-2 total	203.6 ± 38.6	157.3 ± 38.7	3.57	< .001
EDI-2 Drive for thinness	30.05 ± 8.5	15.5 ± 7.9	5.72	< .001
EDI-2 Body dissatisfaction	36.5 ± 10.3	24.6 ± 11.3	3.65	< .001
EDI-2 Bulimia	10.1 ± 2.9	10.8 ± 3.6	0.74	0.46

Note: For each variable mean values ± standard deviation are shown. BDI-II = Beck Depression Inventory–II; BMI = body mass index; BMI-SDS = body mass index standard deviation scores; EDI-2 = Eating Disorder Inventory–2; HC = healthy control; SCL-90-R = Symptom Checklist-90–Revised.

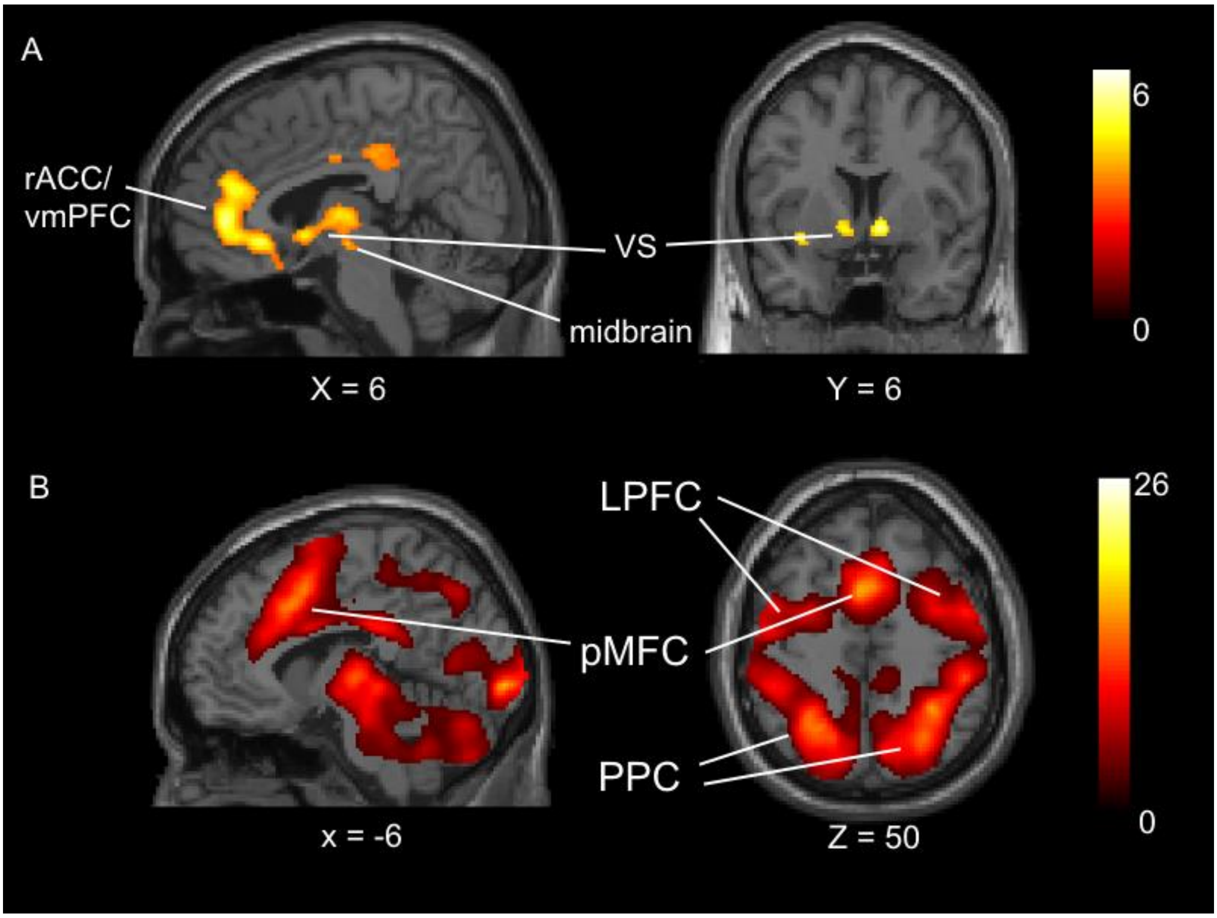

Figure S1: BOLD activation patterns for "value-dependent" (regressor 2) and "value-independent" (regressor 1) contrasts. Familywise error-corrected ($p < 0.05$) T-maps depicting regions in which BOLD activation patterns (A) correlated with subjective value as revealed by a one-sample t-test of the "value-dependent" contrast images (regressor 2) from all longitudinal participants at both time points and (B) generally increased during intertemporal choice as revealed by a one-sample t-test of the "executive decision-making" contrast images(regressor 1) from all longitudinal participants at both time points. Both patterns mirror those found in previous studies which used the same delay discounting paradigm and analysis procedures [23,40,49,52,86,87] (VS=ventral Striatum, vmPFC=ventromedial prefrontal cortex, PCC=posterior cingulate cortex) and (B) activation associated with executive decision making (lPFC=lateral prefrontal cortex, pMFC=posterior medial frontal cortex, PPC=posterior parietal cortex).

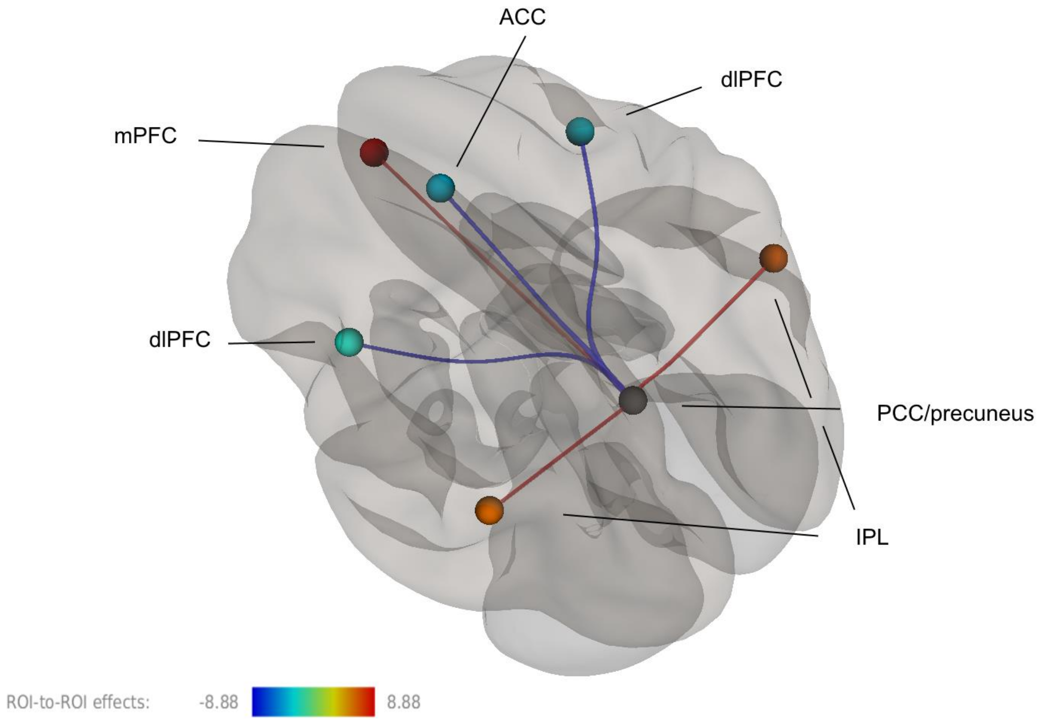

Figure S2: Exemplary 3D plot of the FDR corrected ROI-to-ROI connectivity (RRC) between PCC/precuneus, representing a functional core of the DMN and other DMN regions as well as task-positive regions at TP1 (both extracted from the main analysis). The Fisher-transformed bivariate correlation coefficient between the BOLD time series of two selected ROIs is reported. Anticorrelations are presented in cool colors and positive correlations in warm colors. DMN activity is known to be anticorrelated with activation in so-called task-positive regions that are recruited to meet task demands. Our exploratory functional connectivity analyses confirmed anticorrelated activity between the previously identified DMN regions (mPFC, IPL and IPL) and task-positive brain regions (left and right dorsolateral prefrontal (dlPFC) regions, anterior cingulate cortex (ACC) and thalamus), recruited during the executive decision making phase (regressor1) of our task. No significant longitudinal changes in this connectivity were detected.

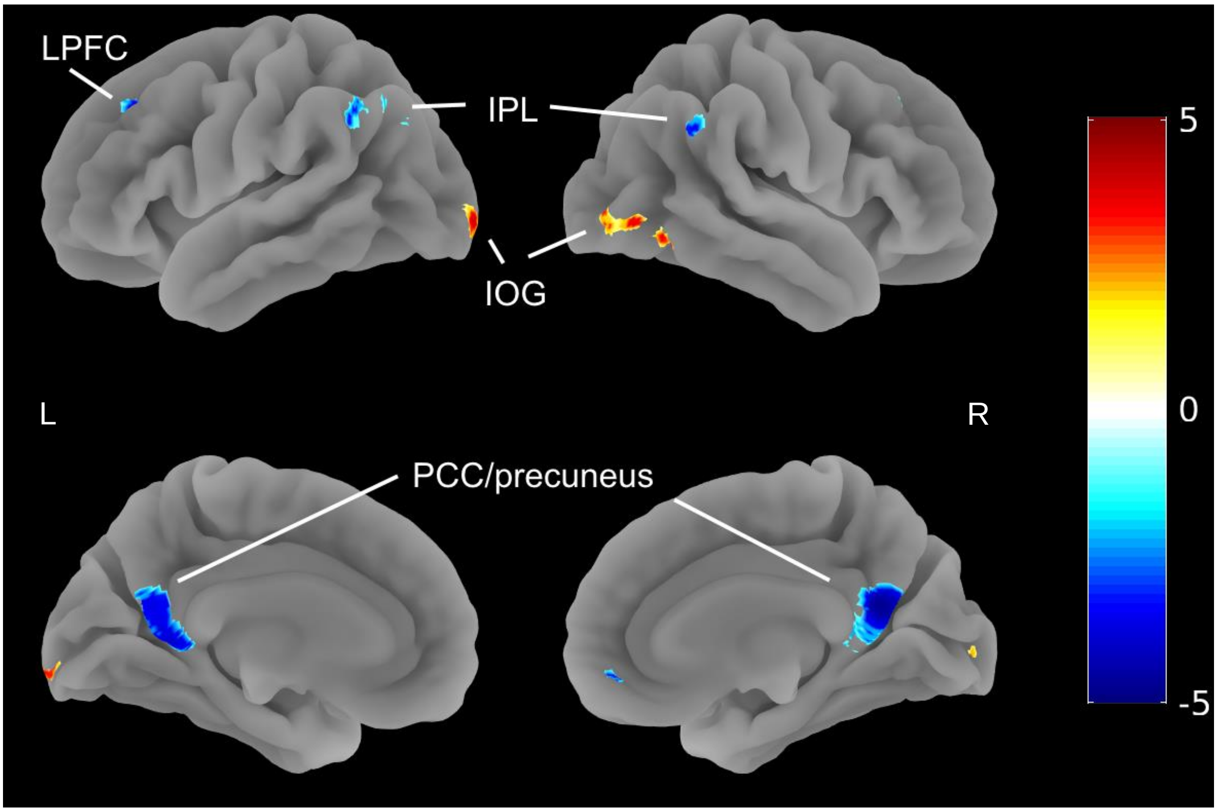

Figure S3. Results of exploratory group comparisons between the acAN-TP1 included in the current longitudinal study (n = 22) and age-matched HC (n =22). While no group differences reached significance in the parametric analysis of value-dependent processing (regressor 2; data not shown), significant group differences in correlates of executive decision making (regressor 1) highly comparable to those found in the larger sample of King et al. (2016) were evident at familywise error-corrected $p < .05$. T-maps illustrate regions with significantly decreased (HC > TP1, cool colors) and increased activation(TP1 > HC, warm colors) in patients relative to controls. Results are projected onto an inflated cortical surface of an individual brain normalized to the Montreal Neurological Institute template. LPFC=lateral prefrontal cortex; IOG=inferior occipital gyrus; IPL=inferior parietal lobe; PCC=posterior cingulate cortex

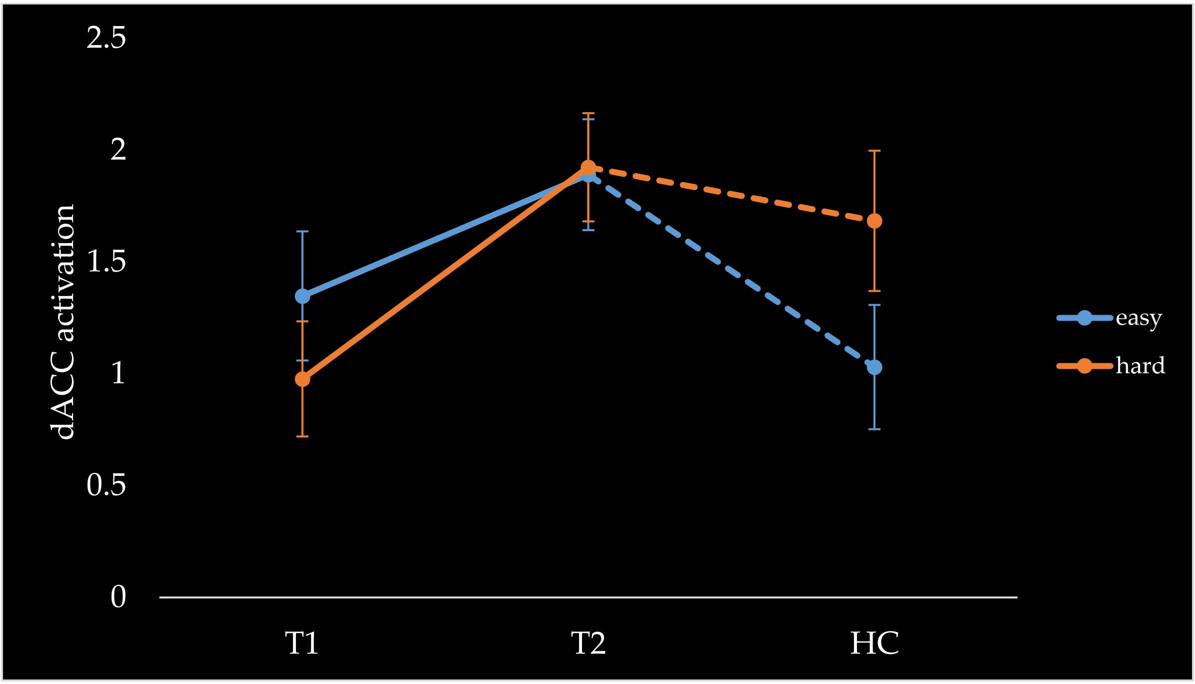

Figure S4: Longitudinal analysis of activation in the dACC ROI identified by King et al. [40]. We report a significant effect of time point between TP1 and TP2 ($F = 8.2$ $p = 0.01$). A tendency for an interaction of group and decision difficulty when comparing HC and acAN TP1 ($F = 3.2$ $p = 0.08$) (comparable to King et al. [40]) and a significant group difference between acAN TP2 and HC ($F = 4.2$ $p < 0.05$). Explorative paired t.tests showed that dACC activity in hard trials increased from TP1 to TP2 ($t_{21} = -3.3$; $p < .05$, and is thus, on a qualitative level, similar to recAN as well as HC), while activity in easy trials did not differ between time points ($t_{21} = -1.3$; n.s.)) [23,40]. Note again the missing longitudinal approach in HC comparison.
